# Sustainable innovation with a method based on peripheral mononuclear cells to screen, monitor and stratify the population at risk of osteoporosis and fractures – a multicenter cross-sectional trial protocol

**DOI:** 10.3389/fendo.2025.1647800

**Published:** 2025-10-02

**Authors:** Francesca Salamanna, Silvia Brogini, Alberto Di Martino, Nicola Baldini, Agostino Gaudio, Pietro Castellino, Deyanira Contartese, Chiara Di Censo, Gianluca Giavaresi, Cesare Faldini, Milena Fini

**Affiliations:** ^1^ Surgical Sciences and Technologies, Istituto di Ricovero e Cura a Carattere Scientifico (IRCCS) Istituto Ortopedico Rizzoli, Bologna, Italy; ^2^ 1st Orthopaedic and Traumatologic Department, Istituto di Ricovero e Cura a Carattere Scientifico (IRCCS) Istituto Ortopedico Rizzoli, Bologna, Italy; ^3^ Department of Biomedical and Neuromotor Science (DIBINEM), University of Bologna, Bologna, Italy; ^4^ Biomedical Science, Technologies, and Nanobiotechnology Lab, Istituto di Ricovero e Cura a Carattere Scientifico (IRCCS) Istituto Ortopedico Rizzoli, Bologna, Italy; ^5^ Department of Clinical and Experimental Medicine, University of Catania, Catania, Italy; ^6^ Scientific Direction, Istituto di Ricovero e Cura a Carattere Scientifico (IRCCS) Istituto Ortopedico Rizzoli, Bologna, Italy

**Keywords:** osteoporosis, peripheral mononuclear cells, *in vitro* diagnostic method, multicenter clinical trial, study protocol

## Abstract

**Introduction:**

Osteoporosis (OP) is a growing global public health challenge, often underdiagnosed and underestimated due to limitations in current diagnostic tools such as DXA-based bone mineral density (BMD) assessment and FRAX score. These methods do not fully capture fracture risk nor account for gender-specific and metabolic differences. A novel patented diagnostic method, based on the *in vitro* behavior of peripheral mononuclear cells (PBMCs) may offer a more accessible, dynamic, and biologically representative approach to OP diagnosis and stratification.

**Methods and analysis:**

This multicenter, double-blind, cross-sectional clinical trial protocol aims to evaluate the diagnostic potential of the PBMC-based test using blood samples (2–5 mL) from 120 participants stratified by BMD (healthy, osteopenic, osteoporotic fractured and non-fractured). PBMCs are isolated and cultured *in vitro* to assess viability, number, size, and spontaneous osteoclast differentiation over time. Additionally, blood samples are analyzed for T lymphocyte subpopulations, pro- and anti-inflammatory cytokines, platelet-related parameters, and markers of bone turnover. All outcomes are analyzed considering gender differences. Correlation and accuracy analyses will determine the relationship between cellular and biochemical markers and OP status.

**Ethics and dissemination:**

The study protocol has been approved by Emilia Romagna’s Ethics Committee (CE-AVEC), Bologna, Italy. Written informed consent is obtained from all participants. Findings of this study will be disseminated through peer-reviewed publications and conference presentations.

**Clinical Trial Registration:**

https://clinicaltrials.gov/, identifier NCT06551155.

## Introduction

Osteoporosis (OP) is a major public health problem, and globally every 30 min one person suffers from an OP fracture, determining direct healthcare costs in Europe of about 37.5 billion (1). Although the World Health Organization (WHO) settles the OP diagnosis by Dual-energy X-ray Absorptiometry (DXA) T-score Bone Mineral Density (BMD) assessment, BMD alone has not enough sensitivity/specificity (2); many OP fractures occur with BMD values above OP diagnostic threshold, and test intervals, accuracy and targeted population are still debated (3). Additionally, Fracture Risk Assessment Tool FRAX, often associated with DXA and interesting for its modest cost, does not reach statistical strength needed to screen and identify OP fractures and stages. In addition to the above-mentioned limits linked to OP definition by DXA T-score, other restrictions exist. OP definition by DXA was developed for Caucasian women, although it has been used for other female populations and for men (4, 5). In addition to the well-known role of estrogen in women, which accelerates bone loss (5), men and women also display structural differences in bone growth, catabolism and size. The larger bone structure of men makes BMD results appear better than what they are (8). Likewise, aging men have more periosteal apposition, less cortical porosity and endocortical resorption than aging women (6). Thus, the same T-scores may mean different fracture risks in men and women of the same age. Additionally, T-scores results, both in men and in women, can be differently affected by the presence of co-morbidities (obesity, osteomalacia, etc). Therefore, BMD alone and/or associated with FRAX cannot represent an accurate predictor of OP and related fracture risk.

Inadequate screening, diagnosis and stage stratification may lead to an increased risk of OP fracture. In this context, advanced methods to support OP stages screening and diagnosis are mandatory (5). The main challenge is to develop tools at low cost and with low power consumption so that low-income countries may also benefit from them. A further requirement is that such technology may be hosted in a laboratory or a portable device; thus, isolated populations could also be assisted. Reliability of quantitative indices to support diagnosis is also important; thus, technology becomes independent of expert examiners’ availability. To try to react to this challenge, in 2018 a European patent has been deposited (IOR European patent n.3008470 March 21, 2018) referred to a method based on simple observation by light microscopy, in which the *in vitro* viability of circulating peripheral blood mononuclear cells (PBMCs) is related with altered bone metabolism and OP (7–9). Furthermore, specific gender related differences in number, dimension, differentiation time and gene and protein expression profiling of PBMCs from OP patients have been observed (9). In the light of these up-and-coming results, this PBMCs-based diagnostic test might complement the existing protocols, giving the possibility to screen and stratify different stages of OP through a common blood collection, feasible simultaneously with routine serum evaluations, also proposing a solution that might reduce gender disparities in OP disease. In this context, the significance of this protocol is to expand and fully develop this patent by a) improving the knowledge and mechanism of actions of the observed PBMCs behavior on which the patented OP tool is based; b) relating the PBMCs *in vitro* behavior with different levels and severity of altered bone metabolism (from osteopenia to bone fragility fractures); c) evaluating the combination of other blood factors measurement linked to OP status (i.e. platelet related parameters, pro- and anti-inflammatory cytokines, lymphocyte subpopulations) to strengthen the reliability of the method. We hypothesize that by answering and highlighting all these unaddressed inquiries this method could represent an advanced and simple strategy to screen, monitor and above all stratify the population at risk of OP and fractures. This test could be done simultaneously and in combination with regular blood tests, without any additional time and with only 2–5 ml of supplementary blood. This advanced method will deliver a safe, effective, sustainable, high-quality, low power consumption, and patient-friendly tool with a maximum health care impact, providing a ‘One-Health’ strategy for OP disease. This claim is supported by the minimally invasive nature of the test (small peripheral blood draw, no radiation), its compatibility with routine laboratory workflows, and its potential scalability to portable, low-power devices.

### Objectives and trial design

#### Primary objective

To analyze the viability, number, size, and differentiation time of PBMCs isolated from peripheral blood samples (2–5 ml) of non-osteopenic and non-osteoporotic patients (hereafter referred to as healthy patients), osteopenic patients, and osteoporotic patients (both fractured and non-fractured) with available DXA or, for fractured patients, DXA prescribed as per clinical practice for secondary prevention.

The aim is to evaluate the sensitivity and specificity of a diagnostic test based on the *in vitro* maintenance and differentiation of PBMCs (European Patent IOR No. 3008470, March 21, 2018) through a multicenter cross-sectional clinical study. Patients are stratified based on their BMD DXA T-score into osteoporotic, fractured and non-fractured (BMD T-score: < -2.5), osteopenic (BMD T-score: -2.5 < T-score < -1), and healthy (BMD T-score: ≥ 1). In accordance with secondary prevention guidelines, patients with a fragility fracture are categorized as ‘osteoporotic fractured’, irrespective of their exact BMD T-score. All assessments are conducted also considering specific gender differences.

#### Secondary objectives

Ø To characterize lymphocyte subpopulations, platelet-related parameters, inflammatory status, and routine biochemical markers of bone turnover in blood samples from patients involved in the multicenter cross-sectional clinical trial. All experiments are conducted considering specific gender differences.
Ø To investigate the relationship between BMD and PBMC properties *in vitro* in terms of viability, number, size, differentiation time, lymphocyte subpopulations, pro-inflammatory cytokines, and biochemical markers of bone turnover, also stratifying by gender, through a correlation study.

This integrative approach aims to overcome the limitations of single-parameter diagnostics, and its reliability will be evaluated through correlation and accuracy analyses, including ROC curves.

## Methods and analysis

### Study setting

The study is a multicenter cross-sectional clinical trial, with activities related to the study performed at two sites (IRCCS Istituto Ortopedico Rizzoli, Bologna, Italy and Azienda Ospedaliero Universitaria Policlinico G. Rodolico-San Marco, Catania, Italy). This trial protocol was produced according to the Standard Protocol Items: Recommendations for Interventional Trials (SPIRIT) reporting guidelines (10).

### Patient and public involvement

Patients contributed to raising research questions by prompting clinicians to find solutions to their BMD and fracture risk. Patients were not involved in planning the design of the study and outcome measures, which were decided according to the field scientific standards.

### Eligibility criteria

Patients are recruited according to the following criteria.

#### Inclusion criteria

Ø Healthy, osteopenic and OP (fractured and unfractured) patients;
Ø Available DXA (hip and/or lumbar spine) BMD T-score;
Ø Men or women > 40 years from two different Italian areas (Bologna, Catania);
Ø Body Mass Index (BMI) between 18.5-29.9 Kg/m^2^;
Ø Informed consent signed.

#### Exclusion criteria

Ø Hematopoietic system diseases;
Ø Bleeding disorders;
Ø Infections (also positive for HIV-HBV-HCV);
Ø Neoplastic pathologies;
Ø State of pregnancy or breast-feeding;
Ø Alcohol use (>20g alcohol per day currently or previously);
Ø Smoking (>10 cigarettes per day currently or previously);
Ø Diabetes.

Smoking and diabetes conditions were excluded to minimize major confounding effects on immune, metabolic, and inflammatory parameters that could interfere with the interpretation of PBMC behavior independently from bone status.

### Intervention

**Figure 1:**
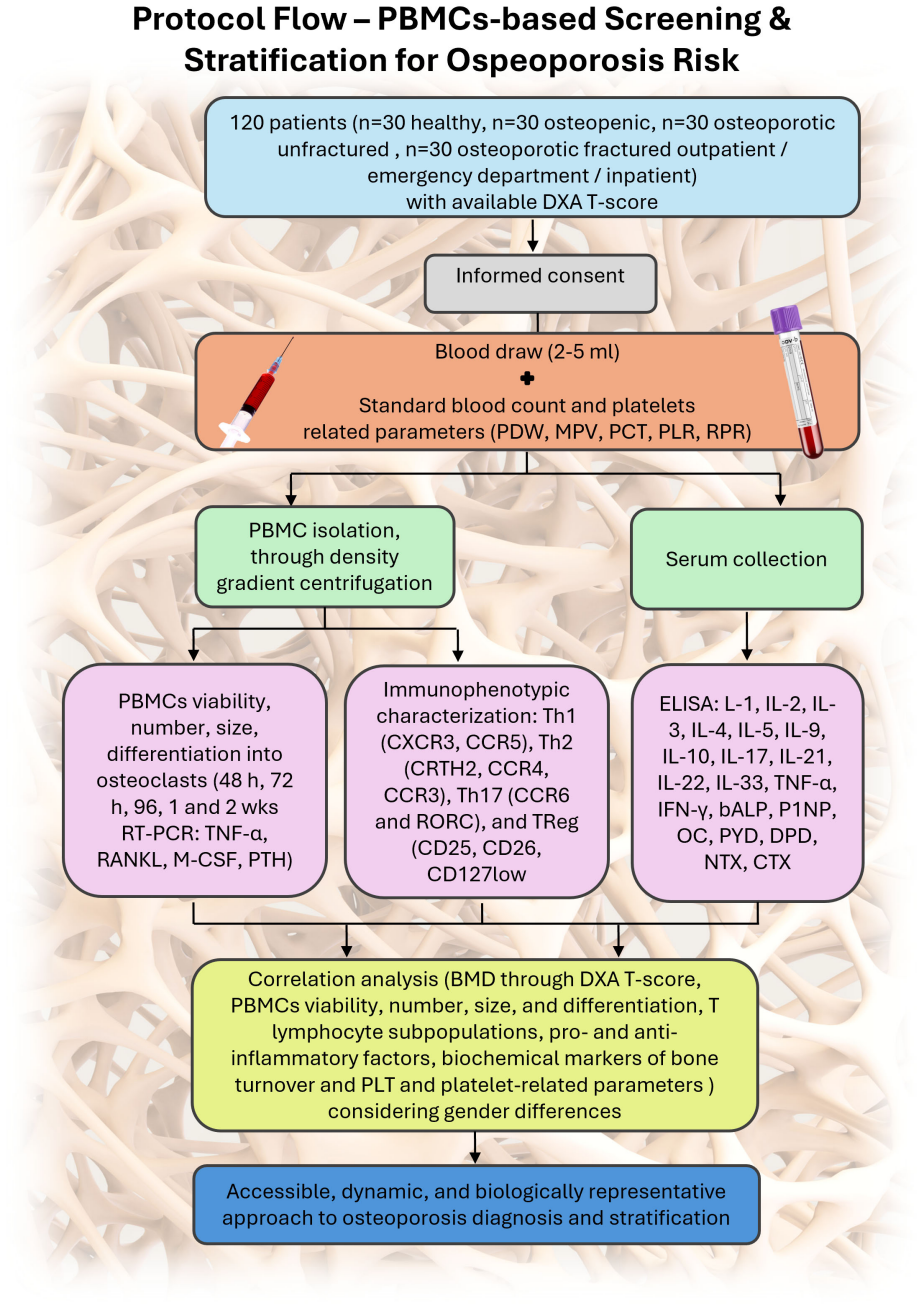
Schematic representation of the study protocol: 120 patients with DXA available or clinically indicated are enrolled. Standard blood analysis is performed alongside a 2–5 mL blood draw used for PBMC isolation and serum collection. PBMCs are divided for immunophenotyping and *in vitro* culture to assess viability, size, spontaneous osteoclastogenesis, and gene expression. Serum cytokines, platelet-related markers, and bone turnover markers are analyzed. Correlation of all variables allows patient stratification.

An overview of the study design and analytical workflow is shown in **Figure 1**. Healthy, osteopenic, and osteoporotic (fractured and non-fractured) patients of both sexes, with available DXA or, for fractured patients, DXA prescribed as per clinical practice for secondary prevention, are enrolled during outpatient visits and/or upon admission to the emergency department and/or hospital, after signing written informed consent. Subsequently, a blood sample (2–5 mL) is collected from each enrolled patient and used for PBMC isolation, performed through density gradient centrifugation, and for serum collection. Once isolated, a portion of PBMCs is cultured *in vitro* (without differentiation factors normally required for PBMCs viability maintenance and differentiation) to assess their viability, number, size, and differentiation into osteoclasts. PBMCs viability are evaluated using optical microscopy and the Alamar Blue assay (a non-toxic reagent that assesses cellular activity through the chemical reduction of resazurin to resorufin in the mitochondria of living cells) at 48, 72, and 96 hours and at 1 and 2 weeks. At the same time points, PBMCs number and size (diameter in µm) are also assessed. Finally, at 2 weeks, spontaneous osteoclastogenesis, i.e., the ability of PBMCs to spontaneously become osteoclasts, is evaluated and quantified through immunocytochemical analysis (Tartrate-Resistant Acid Phosphatase - TRAP - staining) and gene expression analysis *via* RT-PCR (Tumor Necrosis Factor alpha - TNF-α, Receptor Activator of Nuclear Factor κB Ligand – RANKL, Macrophage Colony-Stimulating Factor - M-CSF, Parathyroid Hormone - PTH). Differences in PBMCs viability, number, size, differentiation, and/or activity are also be analyzed considering gender differences. To achieve the secondary objectives of the protocol, a PBMCs aliquot undergoes immunophenotypic characterization to identify the T lymphocyte cell phenotype and evaluate its subpopulations. Specifically, the Th1 (CXCR3, CCR5), Th2 (CRTH2, CCR4, CCR3), Th17 (CCR6 and RORC), and TReg (CD25, CD26, CD127low) subpopulations are analyzed by flow cytometry. Biochemical levels of pro- and anti-inflammatory cytokines are assessed in serum samples from enrolled patients (interleukin (IL)-1, IL-2, IL-3, IL-4, IL-5, IL-9, IL-10, IL-17, IL-21, IL-22, IL-33, TNF-α, Interferon gamma - IFN-γ). Moreover, serum samples are also used to evaluate key markers of bone turnover, including bone-specific alkaline phosphatase, procollagen type I N-terminal propeptide (markers of bone formation) and N- and C-terminal telopeptides of collagen I (markers of bone resorption).

Given the key role of platelets (PLT) and platelet-related parameters in OP, T lymphocyte activation, and pro-inflammatory cytokine production (11), specific laboratory parameters related to platelets, such as PLTs count, Platelet Distribution Width (PDW), Mean Platelet Volume (MPV), Platelet Hematocrit (PCT), Platelet-to-Lymphocyte Ratio (PLR), and Red cell Distribution Width-to-Platelet Ratio (RPR), are also collected and analyzed. All parameters are analyzed considering gender differences.

Finally, to assess the relationship among all analyzed variables—BMD through DXA T-score, PBMCs viability, number, size, and differentiation, T lymphocyte subpopulations, pro- and anti-inflammatory factors, biochemical markers of bone turnover and PLT and platelet-related parameters in blood and serum samples from healthy individuals and osteopenic and osteoporotic (fractured and non-fractured) patients—a correlation study are conducted.

### Expected outcomes

This trial will enable the investigation of PBMCs’ *in vitro* behavior in relation to varying degrees of altered bone metabolism, ranging from osteopenia to fragility fractures. It will also assess correlations with specific blood and serum factors involved in the pathogenesis of osteoporosis, such as T-lymphocytes, platelet parameters, and pro-inflammatory cytokines.

These findings may contribute to enhancing the functionality of the Rizzoli European patent (No. 3008470), including in a gender-specific context, and support the development of the prototype tool already described in the filed patent.

The knowledge gained from this study will provide the National and International Health Care Systems with a safe, functional, high-quality, user-friendly, low-cost, and low-power consumption device, suitable for widespread routine use — particularly in low-income countries.

### Participant timeline

The study has a total duration of 24 months. Ethical approval was obtained on June 20, 2024. Patient enrolment started on 30 August 2024. The conclusion of the study is foreseen before the end of August 2026. After the enrolment, patients undergo blood sampling.

### Recruitment

Healthy individuals, osteopenic patients, and osteoporotic patients (both fractured and non-fractured), of both sexes, are recruited for the study. Eligibility is confirmed based on the availability of a recent DXA scan, or in the case of fractured patients, a DXA prescribed as part of standard clinical practice for secondary prevention. Recruitment takes place during outpatient visits, upon admission to the emergency department, or during hospitalization. All participants are enrolled after providing written informed consent.

### Blinding

This is a double-blind multicenter cross-sectional clinical trial. The application of double-blinding, traditionally associated with interventional studies in which neither participants nor clinicians are aware of key information, typically regarding the assigned treatment, has been adapted in the context of this study to ensure that both laboratory technicians and data analysts remain blinded to the clinical condition of participants. Specifically, the double-blind approach is justified by the following:

- Blinding of laboratory technicians: The laboratory personnel analyzing the biological samples will not have access to information regarding participants’ clinical status. This approach ensures that the laboratory results are obtained objectively, with any observed differences attributable solely to biological variation between groups rather than procedural bias.
- Blinding of data evaluators: The researchers responsible for interpreting the final data will be blinded to the clinical conditions of the participants during the analysis phase. This minimizes the risk of interpretative bias and ensures that conclusions are drawn based solely on objective data.

This blinding strategy, which can be described as a partial double-blind, guarantees that all data are processed with consistency and scientific rigor. It enhances the quality and validity of the findings and aligns with best practices for minimizing subjective influence in biomedical research.

### Allocation

Participants are stratified based on their bone mineral density (BMD), assessed by dual-energy X-ray absorptiometry (DXA) at the hip and lumbar spine. Stratification follows WHO criteria, resulting in three main groups:

- Osteoporotic patients (T-score < -2.5 SD), further divided into fractured and non-fractured subgroups;
- Osteopenic patients (-2.5 SD < T-score < -1 SD);
- Healthy controls (T-score ≥ -1 SD).

A total of 120 participants are enrolled, with 30 individuals allocated to each of the four resulting subgroups (osteoporotic fractured, osteoporotic unfractured, osteopenic, and healthy). Allocation is performed upon confirmation of DXA availability and based on T-score values. No randomization is applied, as group assignment depends on predefined diagnostic criteria.

### Adverse events assessment process

As this is non-interventional no investigational study, treatments or procedures are administered to participants. Therefore, no adverse events directly related to the study protocol are expected. Standard clinical care will be maintained throughout the study period, and any medical events occurring during participation will be managed according to routine clinical practice. Nonetheless, any unexpected or relevant medical issues that arise during study-related procedures (e.g., during blood sampling) will be documented and reported in line with institutional policies and ethical guidelines.

### Data collection and management

Data are collected using a paper-based Case Report Form (CRF). In accordance with the General Data Protection Regulation (GDPR) – EU Regulation 2016/679 – and Clinical Trial Regulation 536/2014, all data within the project are recorded, processed, stored, and managed while ensuring data confidentiality through the implementation of appropriate technical and organizational measures.

### Statistical methods

Statistical analyses are performed using R software (12) and relevant packages, such as ggplot2 (13), for graphical data visualization. A p-value < 0.05 is considered statistically significant.

A total sample size of 120 patients (n = 30 per group) was calculated using G*Power software (power = 0.80, alpha error probability = 0.05). The estimation was based on a Chi-square test (contingency table) with a medium effect size (w = 0.33), derived from the hypothesized difference in PBMC viability (yes/no) between healthy, osteopenic, and osteoporotic patients (fractured and non-fractured). A 20% potential drop-out rate was also considered in the calculation. This sample size ensures adequate statistical power for the primary outcome while maintaining feasibility across the two recruiting centers.

All data are recorded in an electronic dataset comprising continuous variables (e.g., age, PBMC viability, cell count and size, T lymphocyte subpopulation percentages, expression of pro- and anti-inflammatory cytokines, platelet-related parameters, and bone turnover markers, including BMD T-score), nominal categorical variables (e.g., gender, diagnosis), and ordinal categorical variables (e.g., patient stratification). Univariate analyses are conducted to assess the distribution, central tendency (e.g., mean, median), and dispersion (e.g., standard deviation, variance, interquartile range) of quantitative variables, and the frequency distribution of qualitative variables. Continuous variables showing skewed distributions are transformed to approximate normality.

After assessing normality and homogeneity of variances, appropriate statistical tests are applied. For normally distributed data with equal variances, parametric tests such as ANOVA, General Linear Models (GLM), and Student’s t-test are used. In cases of non-normality or heteroscedasticity, equivalent non-parametric tests (e.g., Kruskal–Wallis and Mann–Whitney U tests) are employed.

Patients are stratified by BMD T-score and gender. Correlation, regression, and accuracy analyses are performed accordingly. Specifically, the relationship between BMD and PBMCs viability, count, size, differentiation, T lymphocyte subpopulations, inflammatory cytokines, platelet-related parameters, and serum biochemical markers of bone turnover is investigated. Pearson’s correlation coefficient is used for normally distributed data, and Spearman’s rank correlation is applied for non-parametric data. Receiver Operating Characteristic (ROC) curve analysis is conducted to assess the discriminative power of individual variables.

To avoid information loss and potential selection bias, missing values are imputed. When missingness is minimal, single imputation is used; otherwise, multiple imputations are performed, assuming data are missing at random (MAR) (14). Multiple imputation is conducted using the Multivariate Imputation by Chained Equations (MICE) package in R (15).

### Data monitoring

A central project data manager is responsible for conducting quality control on all collected data. Both an interim report and a final report are submitted to the Italian Ministry of Health, which funded the project (PNRR-POC-2023-12378461).

Monitoring activities are carried out by personnel from the Scientific Directorate and Surgical Science Technology structure of IRCCS Istituto Ortopedico Rizzoli. These units operate independently from the clinical team responsible for conducting the study procedures.

Additional project auditing is performed by the Clinical Trial Centre, an autonomous body within the Institute. The final study report is also submitted to the Ethics Committee for review.

## Ethics and dissemination

### Research ethics approval and consent

Ethical approval was obtained on June 20, 2024, from the Emilia Romagna Ethics Committee (CE-AVEC), Bologna, Italy (protocol number: 342/2024/Sper/IOR). All participants will give informed written consent prior to enrolment. Participants may withdraw from the trial at any time.

### Protocol amendments

Minor amendments to the protocol will be thoroughly documented. Major amendments—such as revisions to the patient information sheet or consent form, changes in the local project leadership, or the addition of a new study site—will be submitted to the Ethics Committee for formal approval.

### Confidentiality and access to data

Data are recorded using CRFs and processed centrally at the IRCCS Istituto Ortopedico Rizzoli, Bologna, Italy. Participants are not identified by name or any other personal data in any study reports, documentation, or files. Instead, each subject is assigned a unique identification code used throughout the study. A static file securely links each code to the corresponding personal information (e.g., name, clinical chart number, telephone number, address). This file, along with all static and dynamic data related to the multicenter, cross-sectional clinical trial—including derived *in vitro* and laboratory data, records, and images—is stored in a password-protected archive created using 7-Zip software.

The encrypted archive is uploaded to a secure cloud server with user access regulated by role-based permissions and protected through single-step login and password authentication. Only the principal investigator and designated key collaborators directly involved in the study are authorized to access individual-level data. Additionally, the Data Protection Officer of IRCCS Istituto Ortopedico Rizzoli creates and stores a backup copy of the archive on a weekly basis. In cases where data exists only in paper format or other non-electronic media, they are scanned, converted into static PDF files, and archived using the same secure procedures applied to electronic data.

### Dissemination policy

This trial protocol has been developed in accordance with the SPIRIT international guidelines. Study results will be disseminated through peer-reviewed publications and submitted for presentation at national and international scientific conferences. To protect patient privacy, raw data will not be publicly shared; access will be restricted to authorized personnel for internal monitoring and data auditing purposes, as well as to the Ethics Committee. Authorship will follow the criteria outlined in the 2018 Recommendations of the International Committee of Medical Journal Editors (ICMJE).

### Scientific relevance and broader impact

OP continues to represent a significant public health issue, primarily due to its strong association with age-related fractures and the substantial socio-economic burden it imposes (16). Conventional diagnostic approaches—such as BMD measurement via DXA and fracture risk assessment tools like FRAX—exhibit notable limitations (17). BMD alone does not adequately reflect the complex and multifactorial nature of fracture risk, while tools like FRAX fail to capture dynamic variables such as rapid bone loss or sex-specific differences in bone architecture (18). Furthermore, existing OP definitions, particularly those relying on T-scores, were originally developed for Caucasian women, and do not account for critical anatomical and metabolic differences between sexes—potentially compromising diagnostic accuracy across diverse populations. This trial addresses these gaps by proposing an innovative, low-cost, and portable diagnostic approach based on the *in vitro* analysis of PBMCs viability from a minimal peripheral blood sample. Although the current research protocol involves extended laboratory steps, the final goal is the development of a simplified diagnostic kit based on automated PBMC viability assays. This will reduce turnaround times compared to those comparable with routine blood tests. The method, developed and patented by IRCCS Istituto Ortopedico Rizzoli, holds the potential to support early diagnosis, disease staging, and risk stratification of osteopenia and OP in a gender-specific and personalized manner. Its ease of use and minimal infrastructure requirements make it particularly suitable for low-resource settings and remote populations, helping to extend screening and monitoring capabilities where they are most needed.

The implementation of this approach through this trial may significantly enhance public health outcomes by enabling earlier intervention, reducing fragility fractures, and improving patients’ quality of life while lowering healthcare costs. Additionally, it paves the way for a robust pipeline of applications—from population-based screening programs to clinical trial stratification and development of research kits—fostering knowledge transfer and collaboration between public and private sectors.

## Strengths and limitations of this study

*-Innovative Diagnostic Approach*.

This study investigates a novel, patented PBMC-based method for OP screening and stratification. By relying on simple blood collection and *in vitro* PBMC behavior, this approach offers a potentially accessible, low-cost, and portable alternative to current diagnostic tools such as DXA and FRAX, which show important limitations.

*-Gender-Specific and Multi-Parameter Evaluation*.

A key strength is the integration of gender-specific analysis and the correlation of PBMC behavior with platelet indices, lymphocyte subpopulations, inflammatory cytokines, and bone turnover markers. This comprehensive profiling may lead to a more precise understanding of OP pathophysiology and risk across different patient subgroups.

*-Double-Blind Design and Multicenter Validation*.

The study design includes a partial double-blind approach to reduce laboratory and interpretation bias, and involves two geographically distinct centers in Italy, strengthening the reproducibility and generalizability of results across different populations.

*-Non-Randomized Design*.

A limitation is the non-randomized nature of the study, which does not allow for assessment of causality or longitudinal changes. While stratification is rigorous, observational bias cannot be entirely ruled out.

*-Restricted Population and Potential Confounders*.

Although several confounding factors (e.g., BMI, comorbidities, substance use) are excluded by design, the inclusion of only patients with recent DXA and a limited age range (>40 years) may reduce generalizability to younger populations or those without DXA access.

## Patient and public involvement

Patients and/or the public were not involved in the design, conduct, reporting or dissemination plans of this research.
